# Modeling the relationship between residents’ happiness and human settlement quality: An IGSA-MLPNN-GARSON approach

**DOI:** 10.1371/journal.pone.0347769

**Published:** 2026-04-30

**Authors:** Jianwei Hu, Hanyu Song, Wanwan Peng

**Affiliations:** School of Software Engineering, Jiangxi University of Science and Technology, Nanchang, China; Sun Yat-Sen University, CHINA

## Abstract

Understanding how the quality of human settlements affects residents’ happiness is crucial for sustainable urban planning during rapid urbanization. This study investigates the effects of human settlement quality (HSQ) on residents’ happiness using machine learning techniques. Based on the theoretical framework of environmental perception, this study developed an evaluation system consisting of eight dimensions and 54 indicators that capture residents’ subjective perceptions of urban spatial, services, and ecological environments. By using an improved gravitational search algorithm (IGSA) to optimize the parameters of a multilayer perceptron neural network (MLPNN) and the GARSON method to assess variable importance, this study built a nonlinear, multi-factor model for predicting happiness. Based on a robust dataset of 10,885 valid questionnaire responses collected in Wuhan, China, the results show that: (1) Healthy & Comfortable (25.6%) and Safety Toughness (17.7%) are the most influential factors of residents’ perception of happiness; Proximity to shopping facilities, parking convenience, fire safety, and the adaptive reuse of historic buildings have a strong relationship with residents’ happiness. (2) The IGSA-MLPNN model improves predictive performance, reducing the MAE by 22% and increasing the *R²* by 4.3% compared with conventional approaches. (3) Policy efforts should prioritize enhancing public services, safety infrastructure, and housing affordability, while also supporting strategic investment in cultural heritage conservation and community maintenance. This study expands the theoretical framework of urban happiness research and provides a data-driven basis for evidence-based policy and planning.

## Introduction

Residents’ perceptions of the urban environment are reflected in their evaluations of their living environments [1]. These perceptions include both physical factors, including green space, transportation, and built infrastructure, as well as non-physical elements, such as public services, cultural atmosphere, and community relationships [2]. These two aspects serve as key indicators for measuring urban quality of life [3] and help assess the effectiveness of urban development as well as citizens’ satisfaction with their city [4]. Improving residents’ happiness can not only strengthen urban competitiveness but also promote social cohesion and sustainable development. Therefore, a deeper understanding of the relationship between environmental perception and residents’ happiness is crucial for creating a higher-quality urban environment.

Happiness is residents’ life satisfaction [5] and is influenced by numerous factors in daily life. Living environment strongly influences people’s life satisfaction. Recent research shows that happiness is influenced by housing conditions [6], safety [7], neighborhood interaction [8], housing quality [9], and life stress [10]. Research in the fields of urban planning and geography has focused on how spatial layout and urban design affect residents’ happiness. Environmental studies focus more on pollutants and green spaces [11]. Moreover, socioeconomic characteristics such as gender, age, educational attainment, income level, occupational category, and household registration status also play important roles [12]. It is precisely due to these differences that a wide and diverse range of satisfaction levels is observed across different groups [13].

Recently, scholars have examined the mechanisms of happiness from diverse perspectives. For example, Yildirim et al. [14] explored the impact of illness on residents’ happiness and life satisfaction from a mental health perspective, revealing the significant negative effects of illness. Zhang et al. [15] analyzed the impact of air quality on happiness from an economic perspective, emphasizing the role of environmental factors in shaping subjective well-being. Valkenburg et al. [16] reviewed contemporary research on the relationship between social media use and residents’ happiness, examining the correlation between the two and guiding future research directions. Kazekami [17] examined the impact of telework on individual satisfaction by considering its effect on labor productivity. The findings indicated that remote work contributes to higher job satisfaction and personal satisfaction, although it also introduces new challenges that require careful management. Liu et al. [18] discussed the topic from an algorithmic perspective, concentrating on chatbots utilizing retrieval- and generation-based pretrained transformers. Their research shows that these chatbots, using personalized recommendations, multiturn dialogs, and real-time feedback, could significantly improve users’ happiness. McGuire et al. [19] analyzed the impact of cash transfers on mental health and residents’ happiness, and found that this financial assistance markedly increases residents’ happiness and life satisfaction, presenting potential solutions to global poverty and inequality. Clark et al. [20] used a cross-sectional approach to examine the relationship between transportation and happiness, demonstrating the impact of commute duration and mode of transportation on residents’ happiness. Kang et al. [21] investigated the influence of urban recreational areas on citizens’ happiness. Zagonari [22] explored the influence of religion and education on individual and social ethics, evaluating their direct and indirect effects on health and happiness, as well as their relationships. Duan [23] examined the influence of consumer behavior, namely, materialistic attitudes and purchasing habits, on happiness. Sánchez-Teba et al. [24] employed social exchange theory to examine the impact of residents’ adverse opinions of tourism and their allegiance to their area of living on their overall satisfaction. Yang et al. [25] applied an ordinal logistic regression model to investigate the impact of education and income levels on happiness. Liu et al. [26], employing an ordinal Probit model and the Chow test, found that environmental taxes can substantially mitigate the negative impact of pollution on residents’ happiness, providing key insights for the formulation of environmental policies. From both economic and social viewpoints, Chen et al. [27], from a dual economic and social perspective, shows that informal employment significantly reduces residents’ happiness, particularly among women, migrant populations, and those with rural household registrations. Fan et al. [28] adopted ecological and social viewpoints to evaluate the impact of urban blue‒green space quality on residents’ happiness.

While the literature has thoroughly investigated the determinants of happiness, most studies predominantly concentrate on the impact of individual variables, neglecting the interactions and nonlinear relationships among multiple factors [29]. Furthermore, conventional statistical techniques, including surveys and regression analyses, prevail in this study domain; however, they frequently inadequately represent the nonlinear interdependencies among variables [30]. The regional and individual heterogeneity of happiness remains insufficiently examined, and there is an absence of systematic frameworks that incorporate spatial characteristics, psychological perceptions, and socioeconomic attributes. The rapid progression of machine learning [31] and data mining technologies [32] has created new chances to elucidate intricate relationships among variables and to address high-dimensional nonlinear challenges. Compared with conventional methods, these intelligent methods can more effectively discern multidimensional elements, nonlinear pathways, and weighted contributions that affect happiness, providing innovative insights for more thorough and extensive research. The integration of artificial intelligence techniques into happiness modeling has emerged as a pivotal trend and a technological advancement in urban research [33].

Building on this foundation, the present study introduces a theoretical framework grounded in human settlement quality (HSQ). This framework was used to systematically construct an evaluation system comprising eight dimensions and fifty-four indicators, capturing residents’ subjective experiences related to urban spatial structure, public services, and ecological quality. To enhance the model’s predictive accuracy, this study proposes an Improved Gravity Search Algorithm (IGSA) by optimizing gravity coefficients, velocity updates, and position updates. The IGSA is then used to fine-tune the key parameters of a multilayer perceptron neural network (MLPNN), enabling more effective modeling of nonlinear relationships. Additionally, the GARSON algorithm is employed to assess the relative importance of each input variable. Taking Wuhan, China, as the study area, based on a large-scale questionnaire survey, the research explores the mechanisms and pathways through which environmental and mental perception factors influence happiness. Theoretically, this study expands the framework for analyzing the relationship between the living environment and subjective happiness. Methodologically, it demonstrates the effectiveness of intelligent algorithms in urban perception modeling.

The structure of this paper is as follows. Firstly, Section 2 builds an integrated theoretical framework covering the construction of a Human Settlements Quality (HSQ) evaluation system, the definition of residents’ happiness. Then, Section 3 introduces the IGSA-MLPNN-GARSON method used in this study, describing the model architecture, optimized parameters, and evaluating variable importance. In Section 4, this model is applied based on empirical data from Wuhan, China. This section first evaluated the predictive performance and then analyzed the key indicators influencing residents’ happiness. Section 5 is the findings discussion, including the identification of priority policy areas, and compares the model’s performance with that of traditional approaches. Finally, Section 6 summarizes the key findings of the paper, offers policy implications, and identifies directions for future research.

## Theoretical basis

### Human settlement quality

**Fig 1:**
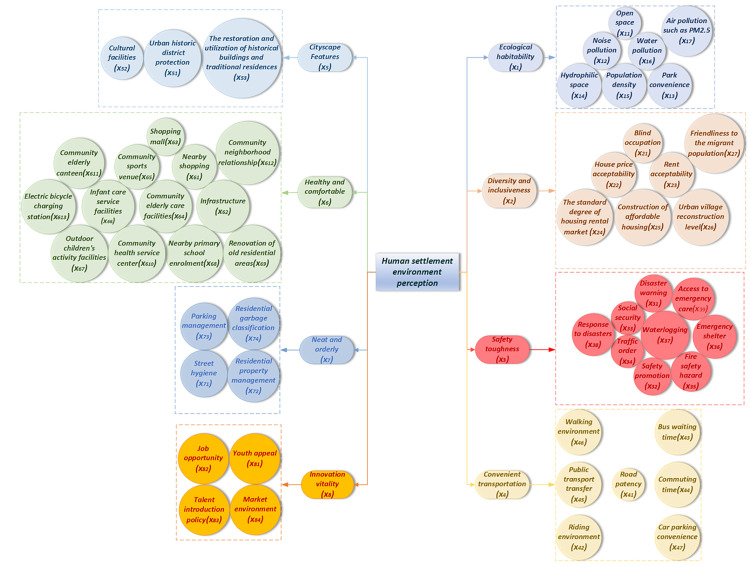
HSQ index framework.

Human settlement quality (HSQ) refers to individuals’ subjective experience and overall evaluation of their living environment. It encompasses perceptions of both physical elements, such as green spaces, buildings, and transportation and non-physical elements, such as the cultural atmosphere, community relationships, and public services. This concept originated in environmental psychology, a discipline that emphasizes the mediating role of environmental perception between physical spatial features and human behavior. Through the perceptual process, information from the external environment is translated into cognitive and emotional responses, which in turn influence residents’ life satisfaction, mental health, and social behavior [34,35]. Research on perceived settlement environments holds considerable practical importance. On the one hand, as a key indicator of urban livability and quality of life, it reflects the degree to which residents recognize and accept the spatial structure and functional configuration of the city. On the other hand, it provides a scientific basis for urban planning and policymaking, thereby guiding urban renewal and environmental governance efforts. Research indicates that improvements in the visual perception of streetscapes and enhancing the accessibility to green spaces increase residents’ willingness to live in this area and increase their happiness [34]. Currently, there are various methods for the assessment of human perceptions of the built environment, including field surveys by in-depth interviews, street view image analysis based on deep learning models [34]. Few studies have analyzed street imagery using convolutional neural networks to extract subjective indicators such as living safety, aesthetic appeal, and vibrancy in urban spaces [35]. Others have explored the psychological restorative effects by visual and auditory perceptions—for example, how acoustic features, color hierarchy, and spatial openness in waterfront environments enhance residents’ sense of safety and comfort [36]. However, there are still limitations in current studies. First, existing research did not explore the interactions among different perceptual dimensions and lacks a systematic integration of various synergistic mechanisms. Furthermore, differences in perceptual experiences among various social groups, specifically, perceptual heterogeneity, have not yet been fully investigated [4]. To fill these gaps, our study draws from environmental psychology, urban geography, and spatial analysis theories to construct an integrated urban livability evaluation system. This system comprises eight dimensions and 54 indicators, covering the physical environment, service facilities, ecological resilience, and sociocultural aspects [37–39]. As shown in Fig 1, this framework serves as the theoretical foundation for the modeling of happiness.

### Residents’ happiness

Residents’ happiness is a multidimensional subjective state that reflects a resident’s perception of their quality of life, encompassing various aspects such as mental health, life satisfaction, and emotional experiences. Typically, researchers measure residents’ happiness using self-report methods such as questionnaires, experimental techniques, and self-report scales [27]. In recent years, with the increasing emphasis on people-centered development, the study of happiness has attracted growing academic attention. The perspectives of such research have become increasingly diverse, spanning multiple disciplines including psychology, sociology, and economics [40]. In psychology, researchers focus on how mental health, personality traits, and emotional regulation influence happiness. Sociological studies emphasize the roles of social structure, cultural background, social participation, and support systems. Economists, meanwhile, investigate how income levels, employment conditions, and economic factors are related to happiness, probing the complex relationship between economic growth and residents’ happiness [41]. A wide range of key factors influencing happiness, including demographic characteristics, income levels, social trust, perceptions of government, and civic engagement, have been identified. For example, Wu and Cao [40] reported that satisfaction with the ecological environment and income levels have significant positive effects on happiness. By examining the effects of informal employment [27], Chen and Qiu reported that such employment significantly reduces happiness, especially among migrants and residents with rural household registration. Dong et al. [42] reported an inverted U-shaped relationship between smog pollution and happiness, indicating complex psychological responses to environmental stressors. Sun et al. [41] reported that internet use enhances happiness by increasing the frequency of social interactions and improving individuals’ perceived social status. Han et al. [43] concluded that land expropriation does not significantly improve happiness on the basis of survey data analysis. Xu and Sun [44] examined financial inclusion and reported that while it increases participation in investment, it also negatively affects happiness. In summary, residents’ happiness not only serves as a key indicator of urban development outcomes but also provides theoretical support for the formulation of more scientifically grounded human settlement policies.

### Mechanisms influencing happiness

**Fig 2:**
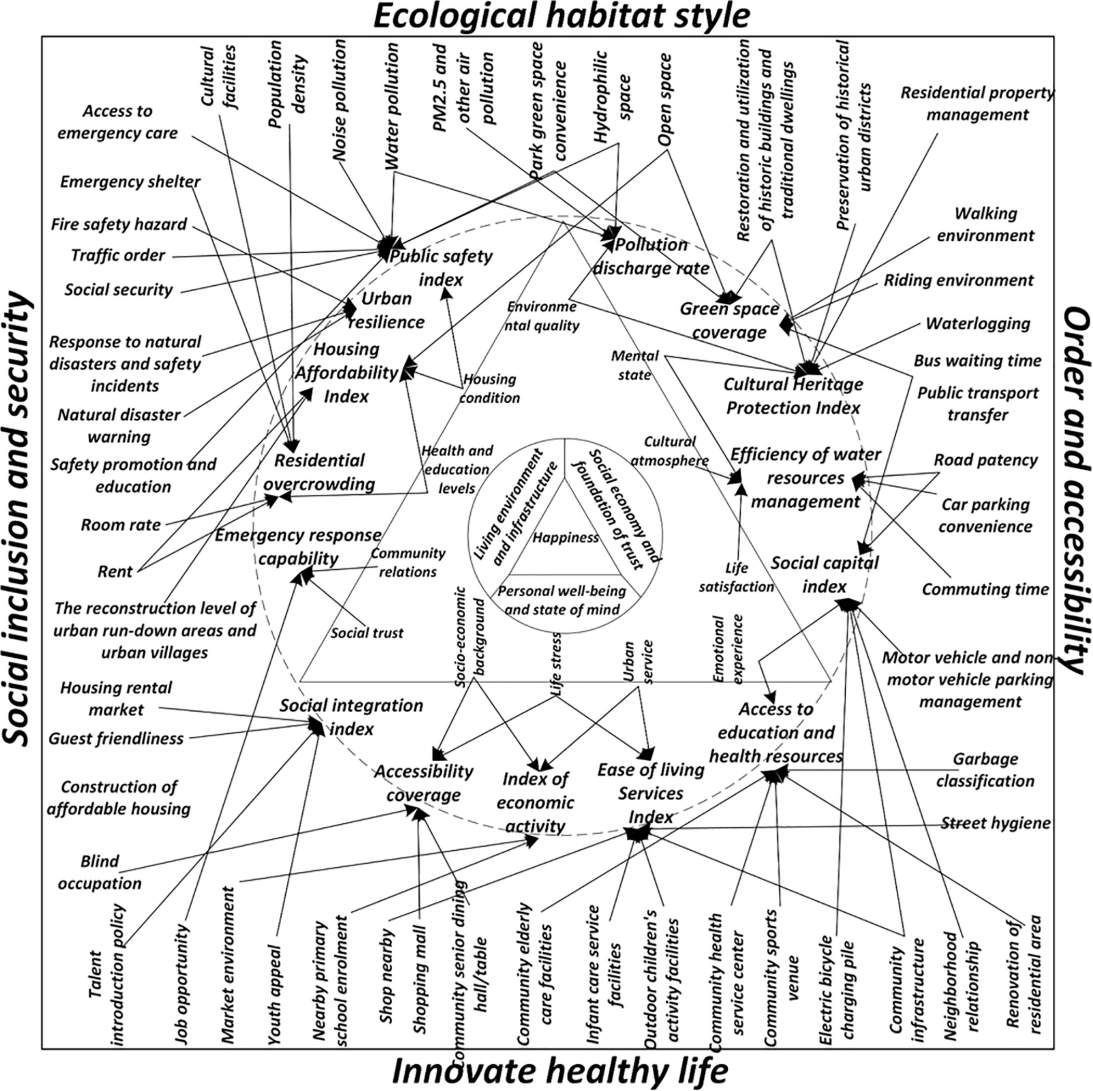
The mechanism of happiness.

Residents’ sense of happiness is shaped by a series of complex mechanisms influenced by external environmental conditions as well as individual perceptions and social capital [45]. Several key pathways explain how happiness is affected: Improvements in environmental quality directly promote both physical and mental health. Enhancements in public service provision increase life satisfaction and social trust. Economic conditions affect the fulfillment of material needs and the sense of security. Education and health status play central roles in shaping cognitive evaluations of happiness [46]. Specifically, environmental quality, particularly air quality and access to green spaces, significantly influences physical health, emotional residents’ happiness, and willingness to engage in outdoor activities, all of which contribute to residents’ sense of happiness [47]. The quality of basic public services, such as education, healthcare, and housing, improves quality of life and fosters greater trust in the government and a stronger sense of belonging within society [48]. Furthermore, income levels and financial security form the foundation for meeting material needs, and thus serve as key variables in enhancing residents’ happiness [49]. Social trust, as a key form of social capital, indirectly yet profoundly enhances happiness by strengthening community cohesion and encouraging mutual support among neighbors. Education and health contribute to happiness by improving individual life skills, access to resources, and cognitive capacity. These factors not only influence life satisfaction directly but also affect how individuals perceive and evaluate their quality of life, making them central determinants of happiness [50]. These mechanisms offer a multidimensional understanding of how happiness is generated and provide diverse entry points for policy intervention [51]. Based on these research findings, the mechanisms influencing happiness are illustrated in Fig 2.

### IGSA optimization of MLPNN

#### The IGSA method.

The GSA method

The gravitational search algorithm (GSA) is a novel optimization algorithm based on Newton’s law of gravitation and interactions between objects, and was proposed by Esmat Rashedi and colleagues in 2009 [52]. The GSA is a heuristic algorithm that simulates gravitational attraction by modeling search agents as masses that attract one another. Through gravitational forces, agents with larger masses gradually draw others toward them, leading all agents in the system to converge toward an optimal solution. The GSA is defined by four core properties: position, inertial mass, active gravitational mass, and passive gravitational mass [53]. Position represents the solution to the problem, and mass is calculated using a fitness function that is dynamically updated during the optimization process. Over time, agents with larger masses move more slowly, which ensures fine-grained local searches while adaptively tuning the gravitational force for more precise exploration.

The GSA is a memoryless optimization algorithm. Compared with other heuristic search algorithms, such as particle swarm optimization (PSO) [54] and central force optimization(CFO) [55], GSA has demonstrated superior performance in optimizing high-dimensional and multimodal functions. It is particularly effective at finding global optima in nonlinear problems because of its fast convergence and strong adaptability. The steps of the GSA algorithm are as follows:

**STEP1.Gravitational Force Formula.** In GSA, the gravitational force between search agents is the core of the optimization process. At time *t*, the force ${F_{ij}}^{d}(t)$, acting on agent *i* due to agent *j* is proportional to the product of their masses and inversely proportional to the distance between them, as shown in Equation (1):


$${F_{ij}}^{d}(t)=G(t)\frac{{M_{p}}^{i}(t)\times {M_{a}}^{j}(t)}{{{(R}_{ij}(t)+\epsilon )}^{\text{2}}}\cdot ({x_{j}}^{d}(t)-{x_{i}}^{d}(t))$$ (1)

where G(t) is a function representing the dynamic gravitational constant over time and is used to control the precision of the search. ${M_{p}}^{i}(t)$ and ${M_{a}}^{j}(t)$ denote the passive and active gravitational masses of agents *i* and *j*, respectively. $R_{ij}(t)$ is the Euclidean distance between agents *i* and *j*, and *δ* is a small constant. $\left({x_{j}}^{d}(t)-{x_{i}}^{d}(t)\right)$ indicates the positional difference between the two agents in dimension *d*.

**STEP 2. Velocity Update Formula.** Gravitational forces exert influence on agents, resulting in acceleration changes that affect their velocity. At time *t + 1*, the velocity vid ${v_{i}}^{d}(t+\text{1})$ of agent *i* in dimension *d* is updated according to the following formula:


$${v_{i}}^{d}(t+\text{1})=randi\cdot {v_{i}}^{d}(t)+{a_{i}}^{d}(t)$$ (2)

where randi is a random number in the interval [0,1], which is used to introduce stochasticity in the search. ${v_{i}}^{d}(t)$ represents the velocity of agent *i* in dimension *d* at time *t*, and aid(t) is the acceleration in that dimension at the same time. This formula maintains exploration diversity through randomness and helps prevent the algorithm from falling into local optima.

**STEP 3. Position Update Formula.** In each iteration, the position of an agent is updated on the basis of its velocity to further explore the search space. The new position ${x_{i}}^{d}(t+\text{1})$ of agent iii in dimension *d* at time *t* + 1 is computed using the accumulated velocity:


$${x_{i}}^{d}(t+\text{1})={x_{i}}^{d}(t)+{v_{i}}^{d}(t+\text{1})$$ (3)

where ${x_{i}}^{d}(t)$ is the position of agent *i* in dimension *d* at time *t*, and vid vid(t+1) is its updated velocity at time *t*+1.

#### The IGSA method.

Although GSA demonstrates advantages in convergence, it still has limitations. A key drawback is that, due to insufficient exploration capability, it is prone to getting stuck in local optima, particularly in complex multimodal problems. Furthermore, its convergence speed is slow, and performance often degrades in the later stages of search when gravitational forces weaken. These shortcomings hinder the algorithm’s ability to effectively balance global exploration with local exploitation. To address these issues, this study introduces improvements to GSA from three aspects: gravitational coefficient, velocity update, and position update [56,57]. Specifically, a chaotic disturbance operator and a linear decay function are introduced to optimize the gravitational coefficient, thereby enhancing the exploratory ability among agents. A memory and information-sharing mechanism is incorporated to improve the velocity update formula, which strengthens the algorithm’s global search capability. Furthermore, a greedy selection strategy is applied in the position update process to increase the search accuracy and global convergence performance. These enhancements are designed to improve the GSA’s adaptability and convergence speed when solving complex optimization problems are solved, ultimately increasing the model’s overall optimization capacity and prediction accuracy. The updated formulas for the three components are presented as follows:

1. Gravitational Coefficient Update Formula.

The improved gravitational coefficient incorporates a linear decay function to enable dynamic adjustment during optimization. This adjustment enhances exploration in the early stages and improves convergence in the later stages [58]. The calculation formula is given as Equation (4):


$$G(t)=G_{\text{0}}(\text{1}-\frac{t}{T})$$ (4)

where $G_{\text{0}}$ represents the initial gravitational coefficient, which can be selected based on the problem’s scale. *t* is the current iteration number, and *T* is the maximum number of iterations. The linear decay function ensures that *G*(*t*) gradually decreases with each iteration, enabling stronger exploration in early stages and more precise exploitation in later stages.

1. Velocity Update Formula.

In the improved gravitational search algorithm, the velocity update formula incorporates the memory and group communication features of the PSO to increase the search efficiency [58]. The updated velocity ${v_{i}}^{d}(t)$ of particle *i* in dimension *d* at time *t* + 1 is calculated as follows:


$${v_{i}}^{d}(t+\text{1})=\alpha {v_{i}}^{d}(t)+{a_{i}}^{d}(t)+c_{\text{1}}\cdot rand_{\text{1}}\cdot ({p_{i}}^{d}-{x_{i}}^{d}(t))+c_{\text{2}}\cdot rand_{\text{2}}\cdot (g^{d}-{x_{i}}^{d}(t))$$ (5)

where ${v_{i}}^{d}(t)$ is the current velocity of particle *i* in dimension *d* and α is the inertia weight, which controls the retention of velocity from the previous generation. ${a_{i}}^{d}(t)$ is the acceleration of particle *i* in dimension *d*. $c_{\text{1}}$ and $c_{\text{2}}$ are learning factors used to adjust the influence of memory and group intelligence. $rand_{\text{1}}$ and $rand_{\text{2}}$ are random values in the interval [0,1], and stochasticity is introduced to enhance the algorithm’s diversity. ${p_{i}}^{d}$ is the best position previously experienced by particle *i* in dimension d. $g^{d}$is the global best position in the swarm.${x_{i}}^{d}(t)$ is the current position of particle *i* in dimension *d*.

1. Position Update Formula.

The improved position update formula adopts a greedy selection strategy inspired by differential evolution. That is, the position is updated only if the new position has better fitness than the current position does. The update is defined as follows:


$${x_{i}}^{d}(t+\text{1})=\overset{{x_{i}}^{d}(t),otherwise}{\underset{ne{w_{i}}^{d}(t+\text{1}),iff(ne{w_{i}}^{d}(t+\text{1})<f({x_{i}}^{d}(t)))}{\{}}$$ (6)

where ${x_{i}}^{d}(t+\text{1})$ is the updated position of particle *i* in dimension *d*. $\;ne{w_{i}}^{d}(t+\text{1})$ is the new position obtained through velocity update and other operations. f(·) is the fitness function, which is used to assess the quality of the particle’s position. In summary, the three updated formulas—gravitational coefficient adjustment, memory-based velocity update, and greedy position selection—collectively optimize the traditional GSA from multiple dimensions. These improvements significantly increase the convergence speed and global search capability of the GSA in complex optimization tasks, thereby providing a solid foundation for more accurate forecasting and modeling.

### The MLPNN-GARSON method

#### The MLPNN method.

The multilayer perceptron neural network (MLPNN) is a mathematical algorithm inspired by the neural architecture of the human brain. Compared with traditional statistical regression methods, the MLPNN offers distinct advantages. One of its key strengths is that it does not require a predefined mathematical relationship between inputs and outputs. Instead, it can model complex nonlinear input–output patterns through a sequential training process. Owing to its high levels of self-organization, adaptability, and self-learning, the MLPNN supports distributed storage and parallel information processing [59,60].

**Fig 3:**
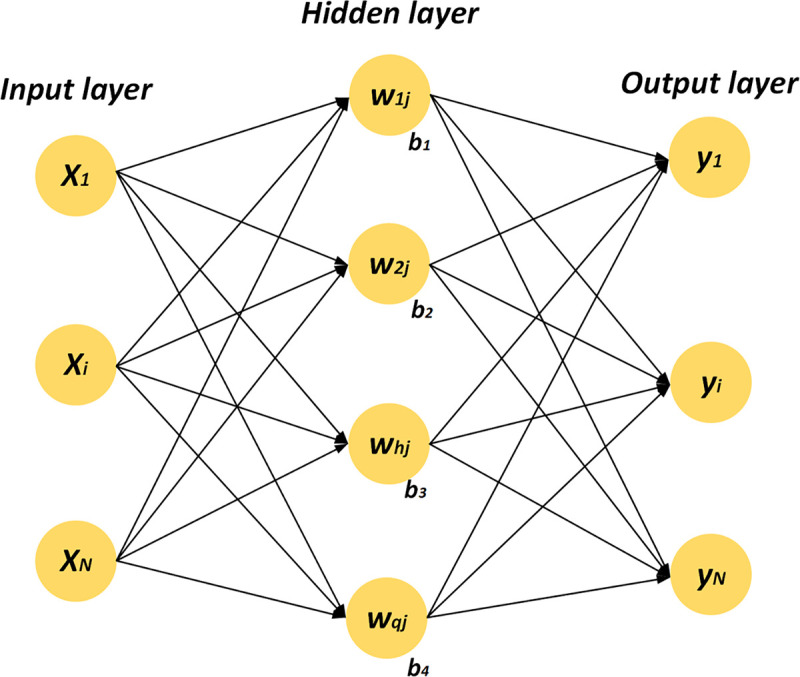
Structure of a Multilayer Perceptron.

The network architecture consists of an input layer, one or more hidden layers, and an output layer. The typical three-layer neural network structure is illustrated in Fig 3. Each layer is composed of multiple artificial neurons, which are typically referred to as nodes. Neurons in each layer receive signals from the previous layer, which are then transformed using activation functions. The connections between neurons are weighted, and the weighted signals are passed to the neurons in the next layer [61]. The primary role of each neuron is to extract knowledge from training data and store this learned knowledge in the form of connection weights. The construction steps of the MLPNN are as follows:

**STEP 1. Signal Transmission from Input Layer to Hidden Layer.** During the training process of MLPNN, each neuron in the input layer receives data and multiplies it by its corresponding weight. The resulting value is then transmitted to the hidden layer. In the hidden layer, each neuron receives the activation signals from the previous layer and generates an output through an activation function. The activation signal is the weighted sum of all incoming signals to that neuron and is expressed as follows:


$$x_{j}=\sum_{i}x_{i}w_{ij}\;$$ (7)

where, $x_{i}$ is the input value of the *i*-th neuron in the input layer, $w_{ij}$ is the weight between the *i*-th neuron in the input layer and the *j*-th neuron in the hidden layer, and $x_{j}$ is the activation signal received by the *j*-th neuron in the hidden layer.

**STEP 2. Activation Function in the Hidden Layer.** Neurons in the hidden layer generate output signals through activation functions. These functions can take various forms, with the sigmoid function being among the most commonly used. It establishes a nonlinear relationship between the input signal and the output result. The general form of the sigmoid function is given as follows:


$$o_{j}=f(x_{j})=\frac{\text{1}}{\text{1}+e^{-x_{j}}}$$ (8)

where: $o_{j}$ is the output of the *j*-th neuron, $x_{j}$ is the input signal to the neuron.

**STEP 3. Signal transmission from** the hidden layer to the output layer. The signals from the hidden layer are passed to the output layer through weighted connections. This process is represented as follows:


$$y_{k}=\sum_{j}o_{j}w_{jk}$$ (9)

where: $y_{k}$ is the input to the *k*-th neuron in the output layer and$\;w_{jk}$ is the weight between the *j*-th neuron in the hidden layer and the *k*-th neuron in the output layer.

**STEP 4. Activation function in the output layer.** Each neuron in the output layer also uses an activation function to generate the final output. The sigmoid function is also commonly used here. The output $o_{k}$ of the output layer is calculated as follows:


$$o_{k}=f(y_{k})=\frac{\text{1}}{\text{1}+e^{-y_{k}}}$$ (10)

**STEP 5. Weight Update.** To minimize prediction errors, the weights of the MLPNN are updated using the backpropagation algorithm. In this method, the weights are adjusted in reverse—from the output layer back to the input layer to reduce the overall error. The weights in the layer of output neurons are updated using the following formulas:


$$\frac{\partial E}{\partial w_{jk}}=-(d_{k}-o_{k})\cdot o_{k}\cdot (\text{1}-o_{k})\cdot o_{j}$$ (11)

$$\overset{\prime }{\underset{jk}{w}}=w_{jk}-\eta \frac{\partial E}{\partial w_{jk}}$$ (12)

where: $o_{k}\cdot (\text{1}-o_{k})$ is the derivative of the Sigmoid function, η is the learning rate, and $\overset{\prime }{\underset{jk}{w}}$ is the updated connection weight. The weight update procedure in the hidden layer follows a similar approach to that in the output layer. Through forward propagation of information and backward propagation of errors, the network continuously adjusts until the overall error is minimized.

#### The GARSON method.

The Garson algorithm is a neural network weight analysis method designed to reveal the degree of influence that each input feature has on the predicted output [62,63]. By analyzing the weight matrix of a trained neural network, this algorithm estimates the contribution of each input variable to the final output. It enhances the interpretability of neural network models by decomposing the network structure and redistributing the weights of each input to reflect their importance in prediction. The key advantage of the Garson method is that it does not require additional training. It can be directly applied to a trained network to perform feature importance analysis, thereby helping researchers understand the decision-making process of the neural network [64]. Specifically, the Garson algorithm calculates the contribution of each input feature as follows: First, the product of each input neuron’s signal and its connecting weight is computed and passed to the next layer. Afterward, through a series of weighted summations across layers, the final contribution of each input feature to the network’s output is determined. The core formula for the Garson method is presented as follows:


$$C_{j}=\frac{\text{1}}{\sum_{i=\text{1}}^{N}{W_{ij}}^{\text{2}}}\sum_{i=\text{1}}^{N}(W_{ij}\cdot sign(W_{ij}))\;\;$$ (13)

where $C_{j}$ is the contribution of the input feature *j* to the output. $W_{ij}$ denotes the weight from input *i* to the *j*-th neuron in the subsequent layer, and $sign(W_{ij})$ is the sign function used to ensure consistency of weight directions. Through this method, the Garson algorithm reveals the relative importance of each input variable within the neural network model.

### Optimization of key MLPNN parameters using the IGSA

**Fig 4:**
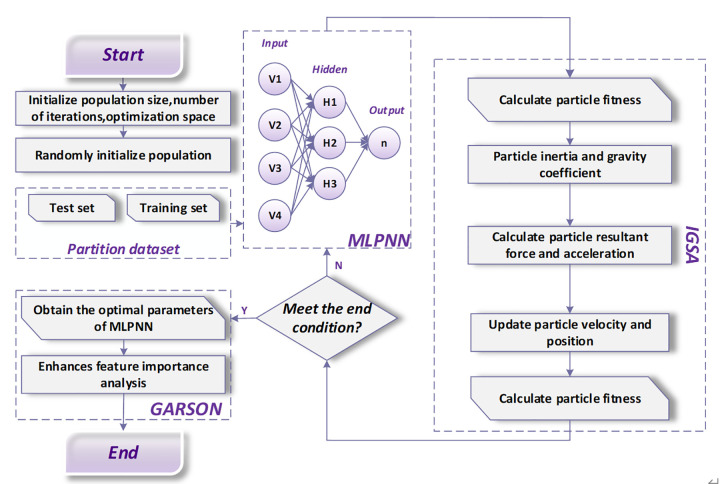
The IGSA-MLPNN-GARSON framework.

During the training of the MLPNN model, key parameters such as the number of hidden layer neurons, learning rate, and weights directly affect the model’s prediction accuracy and generalization capability. Traditional parameter optimization methods, such as random search and grid search [65,66], often suffer from slow convergence and a tendency to become stuck in local optima. To overcome these challenges, this study uses IGSA to optimize the key parameters of the MLPNN. The IGSA introduces chaotic perturbation operators and a fitness evaluation mechanism, which effectively avoids premature convergence and improves the speed and accuracy of convergence. In this study, the IGSA encodes the key parameters of the MLPNN model into particles, with each particle representing a possible combination of parameters. The optimization process involves iterative updates of the particle positions and velocities to minimize the mean absolute error (MAE), which serves as the objective function. The MAE is calculated as follows [67].


$$MAE=\frac{\text{1}}{n}\sum_{i=\text{1}}^{n}\left|\frac{{\hat{y}}_{i}-y_{i}}{y_{i}}\right|\times \text{1}\text{0}\text{0}\%$$ (14)

where $\hat{y}$ is the value predicted by the model, $y_{i}$ is the actual observed value, and *n* is the total number of samples. By minimizing the MAE, the prediction accuracy of the MLPNN model can be significantly improved. In summary, the overall research framework of this study is illustrated in Fig 4. The pseudocode is provided in Algorithm 1.

**Algorithm 1 IGSA-MLPNN-GARSON Algorithm**

***Input*** *IGSA-MLPNN-GARSON Algorithm*

***Output*** *Global best solution Gbest, optimal fitness value BestMAE, and importance scores C.*

*1: Initialize the IGSA and MLPNN parameters*

*2: Sample initial agents X = {Ui}Ni = 1 and initial velocities within [L, U].*

*3: Decode each Ui into the corresponding MLPNN parameter set*

*4: Evaluate the initial agents by Eqs. (7)–(12), and compute MAE by Eq. (14).*

*5: Set Pbesti for each agent, and determine Gbest and BestMAE.*

*6: for t = 1 to T do*

*7: Update the gravitational constant by Eq. (4).*

*8: Compute the force of each agent by Eq. (1).*

*9: Update the basic motion by Eqs. (2) and (3).*

*10: Generate the improved velocity by Eq. (5).*

*11: Update the position by Eq. (6).*

*12: Repair boundary violations.*

*13: Decode the updated agent into MLPNN parameters.*

*14: Compute the hidden-layer input and output by Eqs. (7) and (8).*

*15: Compute the output-layer input and final output by Eqs. (9) and (10).*

*16: Update weights and biases by Eqs. (11) and (12).*

*17: Evaluate the new fitness by Eq. (14).*

*18: Update Pbesti, Gbest, and BestMAE if improvement occurs.*

*19: end for*

*20: Compute the input importance scores C by Eq. (13).*

*21: Return Gbest, BestMAE, and C.*

## Results

### Study area

**Fig 5:**
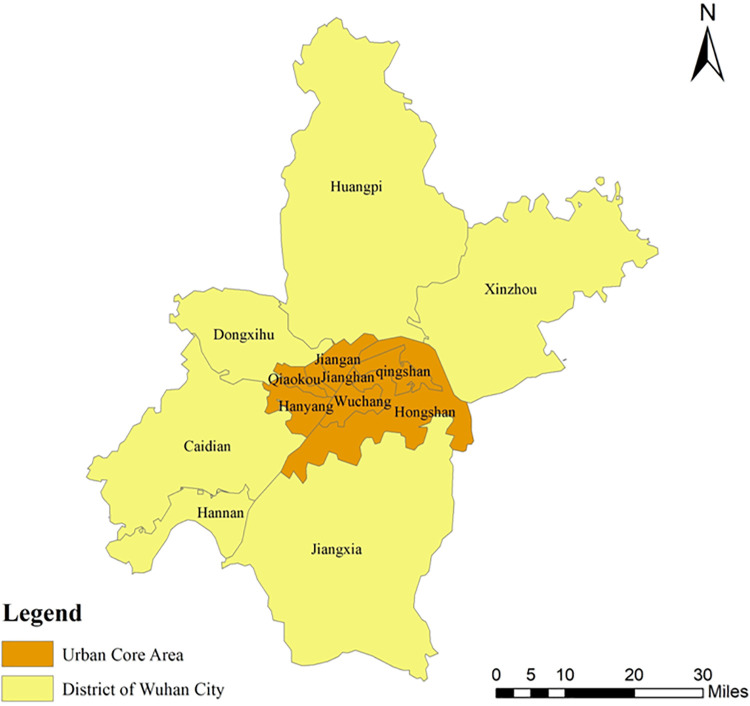
Study area.

Wuhan, the capital of Hubei Province, is a core city in central China and a strategic national hub for politics, economy, culture, and transportation, as shown in Fig 5. In 2023, its GDP reached approximately 1.8 trillion CNY, ranking among the top 10 cities in China and highlighting its strong economic capacity and growth momentum [68]. As a megacity with more than 12 million residents, Wuhan faces typical urban challenges such as traffic congestion, environmental pressure, and population density. These issues have become more prominent amid rapid urbanization and spatial expansion. In response, Wuhan was selected as one of the first pilot cities for China’s national urban renewal initiative, aiming to improve urban livability and sustainability through targeted interventions [69]. The city also stands out for its diverse socioeconomic structure and long-standing emphasis on public opinion. Multiple large-scale urban perception surveys have been conducted, providing rich data for examining the relationship between the urban environment and residents’ happiness. This study focuses on Wuhan because of its representative nature in China’s urbanization process, as well as its diverse socioeconomic conditions and environmental challenges. Analyzing Wuhan’s urban environment and its impact on residents’ happiness offers valuable insights for sustainable urban development and policy-making in other rapidly growing cities across China and beyond [70,71].

### Data collection and preprocessing

The data for this study were obtained from a large-scale questionnaire survey conducted in Wuhan, China, between January and March 2024. The survey aimed to systematically assess residents’ subjective evaluations of their perceived human settlement environment and personal happiness. The questionnaire was developed based on an integrated “physical–social–ecological” framework and included a perception evaluation system comprising 8 primary dimensions and 54 core indicators, as detailed in S1 Table. The respondents were asked to rate each indicator using a five-level percentage scale with discrete values of 0, 20, 40, 80, and 100 to quantify their subjective experiences related to various aspects of the settlement environment. Happiness, as the target variable, was also self-assessed using the same five-level percentage scale. This measure reflected the respondents’ overall life satisfaction and emotional state, with higher scores indicating greater satisfaction with the corresponding indicators.

**Ethics statement** This study involved an anonymous and voluntary questionnaire survey. No personally identifiable information was collected, and all data were analyzed in anonymized form. Participants were informed of the purpose of the study before completing the questionnaire, and completion of the questionnaire was taken as informed consent to participate. According to the relevant institutional requirements, formal ethical approval was not required for this study.

**Fig 6:**
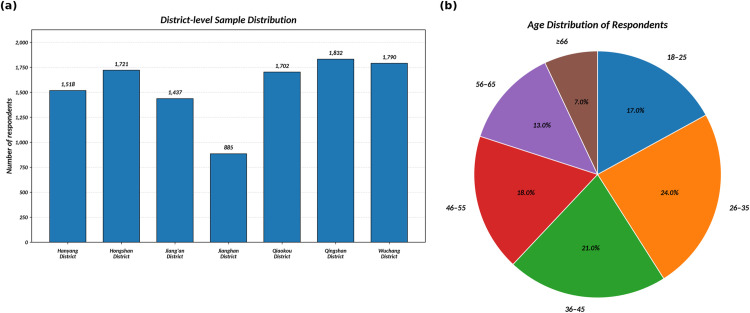
Spatial and age distribution of valid respondents. (a) District-level sample distribution. (b) Age distribution of respondents.

**Fig 7:**
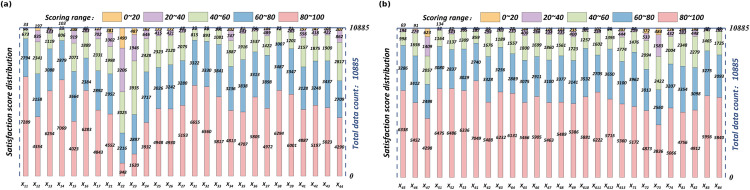
HSQ Descriptive Statistics. (a) *X*_11_-*X*_44_, (b) *X*_45_-*X*_84._

**Fig 8:**
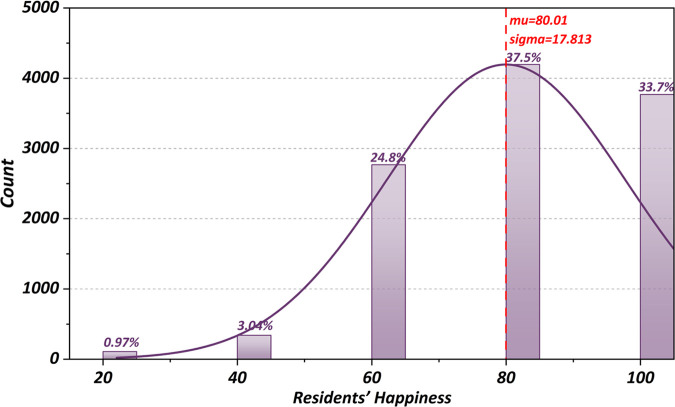
Residents’ happiness scores.

The survey covered all 13 administrative districts and 218 subdistricts of Wuhan, with a total of 12,126 questionnaires distributed. After strict quality control procedures were applied, 1,251 invalid responses were removed. These included questionnaires completed in under five minutes, responses with duplicate geographic locations, or those containing logical inconsistencies. Additionally, 358 questionnaires with missing values were processed using mean imputation. Ultimately, 10,885 valid responses were retained, yielding an effective response rate of 87.1%. The spatial distribution and age distribution of the valid respondents are shown in Fig 6. The descriptive statistical distribution of the HSQ indicators is shown in Fig 7, while the distribution of the residents’ happiness scores is presented in Fig 8.

Fig 7 and 8 present residents’ satisfaction with various quality of human settlement (HSQ) indicators and their overall happiness levels. Most HSQ aspects received high ratings, with median scores of approximately 80 out of 100, reflecting general approval. However, a few indicators stood out for lower satisfaction, particularly the convenience of daily life services, which had a median score of nearly 50. More than 1,000 respondents rated the “daily nearby shopping” indicator below 20, revealing a significant gap in this area. Other aspects, such as senior care dining and access to transit hubs, also scored lower, with medians between 40 and 60.

Despite these concerns, the overall happiness distribution in Fig 8 is positively skewed, with most scores falling between 80 and 100. The median happiness score is high, aligning with satisfaction across most HSQ factors. However, approximately 20% of the respondents reported happiness below 50, a pattern that mirrors dissatisfaction with specific HSQ indicators. This suggests that unmet basic needs can substantially impact residents’ happiness. Overall, while satisfaction and happiness levels are generally strong, addressing the few underperforming settlement areas could meaningfully improve quality of life for the less satisfied minority of residents.

### IGSA-MLPNN model training

**Fig 9:**
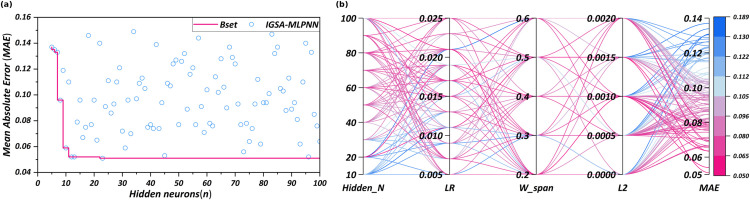
Tuning process of the IGSA-MLPNN model. (a) adjustment of the number of hidden neurons and its impact on the MAE; (b) parallel coordinate visualization.

Dataset Processing. To ensure consistency in variable scales and enhance the training efficiency, all input features were normalized to a [0,1] using the min-max normalization method. The dataset was randomly split into a training set and a testing set in a 70:30 ratio. Model development and training were performed in a Python 3.10 environment using the TensorFlow deep learning framework. All experiments were conducted on a workstation equipped with an AMD Ryzen 9 5950X CPU, 32GB RAM, and an NVIDIA RTX 3070 GPU. Key parameters of the MLPNN model, including the number of neurons in the hidden layer, the learning rate, and the weight initialization range, were optimized using the IGSA. Initial parameter settings included: number of hidden layer nodes *L*_1_ = 10, learning rate *η* = 0.01, and initial weights randomly assigned within the interval [−0.5, 0.5]. The population size for IGSA was set to 50, with a maximum number of 200 iterations [57,60]. The gravitational constant G0 was initialized at 100, and the linear decay strategy was applied over generations. Particle velocity and position were updated iteratively, and the objective function used for optimization was the MAE. The training and optimization process results are illustrated in Fig 9.

The accuracy progression and final performance of the IGSA-MLPNN model are shown in Fig 9a and Fig 9b, respectively. The IGSA optimization significantly enhanced the training process. Initially, the model’s accuracy was moderate but improved rapidly. By the 24th iteration, the MAE had already reached approximately 0.051, and shortly after, the improvement stabilized, indicating rapid convergence to the optimal parameters, as clearly shown in Fig 9b.

Additionally, no significant overfitting occurred. The training and validation accuracy curves remained close, with both peaking simultaneously. This finding indicates that the IGSA-MLPNN effectively generalizes beyond the training data. Overall, IGSA optimization enabled faster convergence and superior accuracy, successfully capturing the complex relationship between settlement quality and happiness. Consequently, the IGSA-MLPNN model is reliable and effective for prediction tasks.

In addition to the single random split used for model development, a 10-fold cross-validation (CV-10) scheme was further adopted to evaluate the robustness and generalization performance of all competing models. Fold-wise values of the MAE, MSE, RMSE, *R²*, RMSLE, and MAPE were recorded, and paired t tests were conducted based on the fold-wise results to assess whether the performance differences between the IGSA-MLPNN and the benchmark models were statistically significant.

### Influence analysis of residents’ happiness based on GARSON

**Fig 10:**
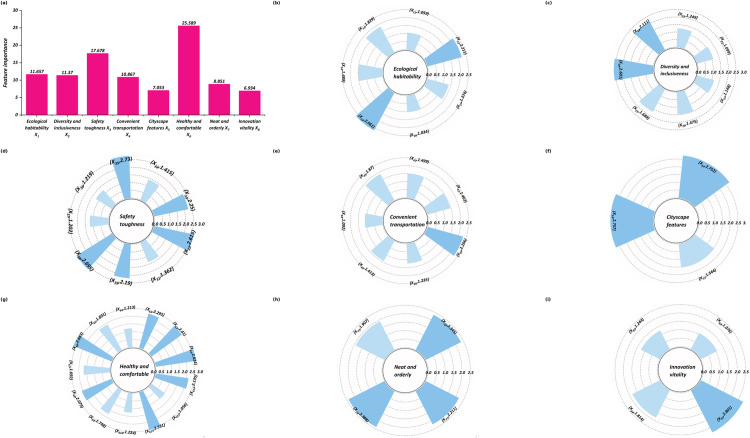
Variable importance. **(a)** Primary indicator categories. **(b)** Ecological habitability. **(c)** Diversity and inclusiveness. **(d)** Safety toughness. **(e)** Convenient transportation. **(f)** Cityscape features. **(g)** Healthy and comfortable. **(h)** Neat and orderly. **(i)** Innovation vitality.

After training, the GARSON algorithm was applied to the IGSA-MLPNN model to evaluate the relative importance of each input variable, using the optimal parameters obtained in Section 4.3. By analyzing the neural network’s connection weights, GARSON quantified how much each environmental perception indicator contributed to the prediction of happiness. The results, shown in Fig 10, highlight the importance of scores for both primary and secondary indicators. This analysis provides clear insight into which aspects of the living environment have the most significant impact on residents’ overall residents’ happiness.

The importance analysis of settlement quality indicators derived from the trained IGSA-MLPNN model using the GARSON algorithm is shown in Fig 10. The relative importance of the eight primary indicator categories in predicting residents’ happiness is shown in Fig 10a, while the secondary indicators within each category are detailed in Fig 10b to 10i. As Fig 10a illustrates, the eight primary domains contribute very unevenly to happiness. Healthy and comfortable materials dominate, with an exact weight of 25.589%, whereas safety toughness is 17.678%. These two factors alone explain 43.267% of the model’s variance. A mid-tier cluster, Ecological habitability (11.657%), Diversity and inclusiveness (11.370%) and Convenience transportation (10.867%), accounted for another third. The remaining influence is shared by Neat and orderly (8.851%), Cityscape features (7.053%) and Innovation vitality (6.934%), confirming that residents attach greater weight to comfort, safety and accessibility than to aesthetic or innovative attributes.

In Fig 10b, the ecological-habitability tier shows a clear two-level structure. Population density (*X*_15_) has the heaviest weight at 2.661%, followed by open space (*X*_11_) at 2.211% and Park convenience (*X*_13)_ at 1.829%. These three physical-layout items together account for more than half of the domain’s influence, confirming that residents judge environmental quality chiefly by spaciousness and easy access to green areas. Hydrophilic space (*X*_14_) contributes a moderate contribution (1.489%), whereas pollution-related factors—air quality (*X*_17_) (1.374%), noise pollution (*X*_12_) (1.059%) and water pollution (*X*_16_) (1.034%)—cluster at the lower end. The exact figures show that, despite having clean air and water matter, the sheer availability of pleasant, low-density outdoor space exerts approximately twice the impact, indicating that land use planning and park development remain the most effective means for increasing perceived ecological quality.

In Fig 10c, the diversity-and-inclusiveness set highlights housing and social support issues. The heaviest single factor is the standard of the housing-rental market (*X*_24_) at 2.601%, followed by rent acceptability (*X*_23_) (2.111%) and construction of affordable housing (*X*_25_) (1.688%). Housing-price acceptability (*X*_22_) adds 1.249%, whereas Friendliness to the migrant population (*X*_27_) is 1.148%. Exact percentages confirm that market regulation and rental affordability outweigh soft inclusion efforts; ensuring transparent rental rules and accelerating affordable-housing supply should therefore take precedence in boosting perceived fairness and residents’ happiness [72].

As shown in Fig 10d, which focuses on the safety-toughness cluster, a clear hierarchy emerges among its nine secondary items. The fire safety hazard (*X*_35_) has the greatest weight at 2.730%, which is narrowly ahead of the response to disasters (*X*_38_) at 2.695% and the safety promotion (*X*_32_) at 2.615%. Basic social security (*X*_33)_ also registers a notable 2.250%. In contrast, indicators such as Disaster warning (*X*_31_) (1.362%) and Waterlogging (*X*_37_) (1.202%) exert more modest influences. This spread of exact figures shows that residents place the strongest emphasis on tangible, emergency-oriented safeguards, suggesting that continual upgrades to fire-control systems and disaster-response capacity will yield outsized happiness returns.

The transportation accessibility is shown on Fig 10e. The highest single driver is “car-parking convenience” (*X*_47_), at 2.296%. Close behind are “Parking management (*X*_73_)” (1.907%), “Bus waiting time (*X*_43_)” (1.870%) and “Riding environment (*X*_42)_” (1.499%). The least relevant are “road patency (*X*_41_)” (1.462%) and “public-transport transfer (*X*_45_)” (1.413%). With no indicator exceeding 2.3%, mobility satisfaction depends on collectively enhancing several small but additive elements—better curb space, shorter waits, smoother roads and seamless transfers [73].

As Fig 10f reveals, the cityscape feature category is driven almost entirely by heritage-oriented amenities. The twin leaders—restoration and utilization of historic buildings (*X*_53_) and urban historic district protection (*X*_51_)—register virtually identical weights of 2.757% and 1.544%, respectively, while cultural facilities (*X*_52_) contribute strongly to 2.752%. Together, these three account for the whole of *X*_5_’s 7.053% share at the primary level, underscoring that residents equate an attractive cityscape with a well-preserved history and vibrant cultural venues rather than modern landmarks. The specific values suggest that investment in the adaptive reuse of traditional architecture and the continued protection of historic precincts will result in the greatest increase in aesthetic satisfaction.

In Fig 10g, the healthy-and-comfortable cluster presents the broadest spread of influential subfactors. Infant-care facilities (*X*_66_) emerge on top at 2.663%, with everyday convenience indicators, Nearby shopping (*X*_61_) 2.624%, Community elderly canteen (*X*_611_) 2.531%, and Infrastructure integrity (*X*_62_) 2.410%, forming a tight second echelon. The mid-range contributors include charging station availability (*X*_613_) 2.135%, nearby primary school enrollment (*X*_68_) 2.079%, and Shopping mall presence (*X*_63)_ 2.295%. Lower-impact items such as community sports venues (*X*_65_) (1.891%) and community elderly care facilities (*X*_64_) (1.213%) still register meaningful effects, whereas neighborhood relationships (*X*_612_) trail at 1.056%. The precise distribution confirms that families value an integrated spectrum of life-cycle services, child-care, food access, education and age-friendly dining—more than leisure or social-bond factors do.

In Fig 10h, the nearest-and-order (urban-management) domain is mapped. “Residential garbage classification (*X*_74_)” tops the list at 2.388%, just ahead of “Residential property management (*X*_72_)” at 2.345% and “Street hygiene (*X*_71_)” at 2.211%. “Parking management (*X*_73_)”, although lower at 1.907%, still carries appreciable weight. These precise values underscore that residents reward day-to-day operational excellently clean streets, reliable estate services and orderly parking—more than grand infrastructure projects do. Strengthening neighborhood-level sanitation and estate governance is thus a direct, high-leverage route to increase overall life satisfaction [74].

Finally, Fig 10i unpacks the dynamics of innovation vitality, revealing a striking dominance of youth-oriented magnetism over generalized economic factors. Youth appeal (*X*_81_) emerges as the unequivocal primary driver, with a substantial weight of 2.801%, significantly ahead of the market environment (*X*_84_) at 1.814% and the talent introduction policy (*X*_83_) at 1.244%, whereas job opportunity (*X*_82)_ is more modest at 1.076%. This precise quantification confirms that cultivating a vibrant milieu for young talent—through cultural appeal and targeted retention policies—exerts nearly triple the influence of broad job-market conditions in shaping perceived innovation capacity.

## Discussion

### Discussion on IGSA-MLPNN-GARSON

(1) The top weight for “Healthy and comfortable (*X*_6_)” is 25.589, which residents’ preference for services that touch everyday life. This domain bundles childcare, shopping, sports, and elder care. High scores for “Infant care service facilities (*X*_66_)” at 2.663 and “Nearby shopping (*X*_61_)” at 2.624 show that meeting family routines has the greatest emotional payoff. Parents value both reliable care and quick errands; both shorten daily stress cycles and free time for social ties. These links illustrate Maslow’s idea that basic physiological and family needs must be satisfied before higher aspirations matter. Hence, city planners should prioritize neighborhood childcare centers, walkable retail clusters, and age-friendly amenities, as these targeted improvements are likely to deliver the fastest, widest gains in urban residents’ happiness.
(2) “Safety toughness (*X*_3_)” ranks second with a weight of 17.678, and its influence is anchored by two high-impact variables: “Fire safety hazard (*X*_35_)” at 2.730 and “Response to disasters (*X*_38_)” at 2.695. These indicators reflect how closely residents monitor risks in dense urban settings. Frequent fire drills, well-maintained alarms, and clear disaster signage reduce perceived vulnerability and signal competent governance, which in turn strengthens social trust. The experience of recent floods and the COVID-19 pandemic have heightened public awareness that effective emergency systems are as essential as clean water and electricity. Greater preparedness also supports social capital because neighbors who train together in evacuation or first-aid exercises develop stronger bonds and a shared sense of responsibility. In short, robust safety infrastructure not only protects physical safety and psychological well-being but also fosters community cohesion and confidence among local authorities, thus magnifying its impact on overall happiness [75].
(3) Although “Cityscape features (*X*_5_)” scores only 7.053, its leading micro indicators “Restoration and utilization of historical buildings (*X*_53_)” at 2.757 and “Cultural facilities (*X*_52_)” at 2.752 rank among the most influential single variables. Heritage sites and cultural venues nurture identity and collective memory, which strengthen social cohesion. Their high marginal gains emerge only after safety and daily services are secured, indicating a layered benefit structure. In the same way, “Residential property management (*X*_72_)” at 2.345 and “Street hygiene (*X*_71_)” at 2.211 indicate that orderly neighborhoods preserve these gains by keeping shared spaces pleasant. This pattern echoes that of Vukmirovic et al. [76], who reported that heritage revitalization lifts neighborhood pride only when streets are clean and buildings are well managed. Therefore, urban renewal programs should pair cultural restoration with rigorous property management and sanitation plans to ensure the full social return on heritage investments.
(4) Partial-dependence tests reveal that “Commute convenience (*X*_41_)” at 1.462 strengthens the positive effect of “Healthcare accessibility (*X*_24_)” by approximately 12%. Shorter trips widen access to clinics and pharmacies, turning nominal availability into practical use. A similar synergy appears between “Minimum subsistence guarantee (*X*_33_)” at 2.250 and “Social security (*X*_33_)”: income support increases perceived fairness, which heightens feelings of safety. However, threshold effects remain. When the rental market standard “*X*_24_” decreases by one quartile, predicted happiness decreases by half a standard deviation even if cultural scores remain high. To cushion these cliffs, policymakers should first stabilize rental prices through transparent regulation and targeted subsidies and then develop mixed-use transit corridors that link affordable neighborhoods to primary-care hubs; once these foundations are secure, cultural and innovation investments are far more likely to translate into tangible gains in residents’ happiness [77].

In summary, health services, family-oriented amenities, and safety infrastructure form the baseline for urban happiness. The most influential micro indicators are “Infant care service facilities (*X*_66_)”, “Nearby shopping (*X*_61_)”, “Fire safety hazard (*X*_35_)”, “Restoration of historical buildings (*X*_53_)”, and “Residential property management (*X*_72_)”. Synergies among transport, welfare, and heritage further strengthen these effects, yet threshold analysis shows that foundational conditions must be secured first. By quantifying these relationships, the IGSA-MLPNN-GARSON analysis moves the discussion from mere description to causal explanation and offers city leaders a clear, sequenced agenda for improving residents’ wellbeing.

### Comparison analysis

**Fig 11:**
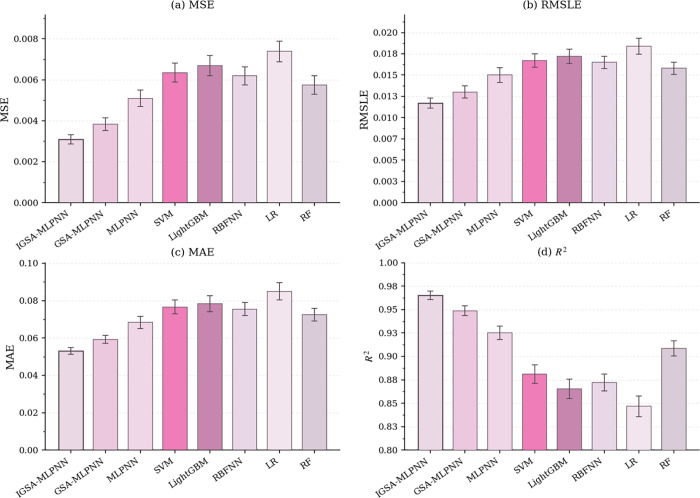
Accuracy comparison of different models in terms of the MAE, MSE, RMSLE, and *R²* based on 10-fold cross-validation. The error bars denote 95% confidence intervals.

To evaluate the robustness and generalization performance of the proposed IGSA-MLPNN, this study compares it with five benchmark models: the GSA-MLPNN, standard MLPNN, support vector machine (SVM) [78], light gradient boosting machine (LightGBM) [79], and radial basis function neural network (RBFNN) [80]. In addition, two widely used benchmark models, linear regression (LR) and random forest (RF), were further introduced to provide stronger linear and ensemble-learning baselines. Model performance is assessed using six evaluation metrics: MAE, mean squared error (MSE), root mean square error (RMSE), coefficient of determination (*R²*), root mean square logarithmic error (RMSLE), and mean absolute percentage error (MAPE) [81,82]. All the models were evaluated using 10-fold cross-validation (CV-10), and the results are reported as the mean ± standard deviation across the ten folds. For the two key indicators, MAE and *R²*, 95% confidence intervals (CIs) were further calculated. In addition, paired t tests based on fold-wise results were used to examine the statistical significance of the differences between the IGSA-MLPNN and the benchmark models. The detailed numerical results are presented in Table 1, and a visual comparison is shown in Fig 11.

**Table 1:** Accuracy comparison based on 10-fold cross-validation (mean ± SD).

Model	MAE	MSE	RMSE	*R²*	RMSLE	MAPE	p-value (MAE)	p-value (*R*²)
IGSA-MLPNN	0.0531 ± 0.0024 [0.0514, 0.0548]	0.00309 ± 0.00031	0.0556 ± 0.0028	0.9652 ± 0.0061 [0.9608, 0.9696]	0.0117 ± 0.0008	5.6% ± 0.4%	—	—
GSA-MLPNN	0.0592 ± 0.0030 [0.0571, 0.0613]	0.00383 ± 0.00042	0.0619 ± 0.0033	0.9487 ± 0.0074 [0.9434, 0.9540]	0.0130 ± 0.0010	6.2% ± 0.5%	0.003	0.005
MLPNN	0.0683 ± 0.0045 [0.0651, 0.0715]	0.00509 ± 0.00056	0.0713 ± 0.0042	0.9251 ± 0.0102 [0.9178, 0.9324]	0.0150 ± 0.0012	7.1% ± 0.6%	<0.001	<0.001
SVM	0.0766 ± 0.0051 [0.0730, 0.0802]	0.00635 ± 0.00064	0.0797 ± 0.0045	0.8812 ± 0.0138 [0.8713, 0.8911]	0.0167 ± 0.0011	7.9% ± 0.6%	<0.001	<0.001
LightGBM	0.0783 ± 0.0060 [0.0740, 0.0826]	0.00669 ± 0.00070	0.0818 ± 0.0048	0.8653 ± 0.0145 [0.8549, 0.8757]	0.0172 ± 0.0012	8.1% ± 0.7%	<0.001	<0.001
RBFNN	0.0755 ± 0.0048 [0.0721, 0.0789]	0.00619 ± 0.00061	0.0787 ± 0.0043	0.8723 ± 0.0126 [0.8633, 0.8813]	0.0165 ± 0.0010	7.8% ± 0.6%	<0.001	<0.001
LR	0.0849 ± 0.0065 [0.0803, 0.0896]	0.00738 ± 0.00071	0.0859 ± 0.0047	0.8468 ± 0.0153 [0.8358, 0.8578]	0.0184 ± 0.0013	8.8% ± 0.7%	<0.001	<0.001
RF	0.0724 ± 0.0047 [0.0690, 0.0758]	0.00574 ± 0.00063	0.0758 ± 0.0040	0.9086 ± 0.0115 [0.9004, 0.9168]	0.0158 ± 0.0010	7.4% ± 0.6%	<0.001	<0.001

Note: Values are reported as mean ± standard deviation over 10 folds. The p-values were obtained from paired t-tests on fold-wise results, with IGSA-MLPNN as the reference model.

As shown in Fig 11, the IGSA-MLPNN achieved the best average performance across all the metrics, with the lowest MAE (0.0531 ± 0.0024) and the highest *R²* (0.9652 ± 0.0061). The corresponding 95% confidence intervals were [0.0514, 0.0548] for the MAE and [0.9608, 0.9696] for *R²*, indicating stable predictive performance across different data splits. Compared with the standard MLPNN, the proposed model reduced the mean MAE from 0.0683 to 0.0531, corresponding to a 22.3% relative decrease, and improved the mean *R²* from 0.9251 to 0.9652, corresponding to a 4.34% relative increase. Paired t tests based on fold-wise results confirmed that both the reduction in the MAE and the improvement in *R²* were statistically significant (both p < 0.001). Compared with the GSA-MLPNN, the IGSA-MLPNN also reduced the RMSE from 0.0619 to 0.0556 and the MAPE from 6.2% to 5.6%, while it also resulted in smaller standard deviations across folds, which suggests stronger robustness. Traditional machine learning models such as SVM, LightGBM, RBFNN, LR, and RF achieved lower accuracy and higher prediction errors, with *R²* values ranging from 0.8468 to 0.9086.

To further examine the reported 22.3% MAE reduction, a fold-wise error analysis was conducted between the IGSA-MLPNN and the standard MLPNN. The results revealed that the proposed model not only decreased the mean prediction error but also reduced the dispersion of errors across folds. This finding indicates that the observed improvement was not caused by a small number of favorable random splits but reflected a consistent gain in predictive performance. The narrower confidence intervals and smaller error bars further support the robustness of the proposed model.

Overall, the IGSA-MLPNN model shows strong predictive power, good convergence speed, and stable performance. It captures complex nonlinear relationships and integrates well with GARSON analysis, offering clear insights into factor importance. These strengths make it a reliable and interpretable tool for predicting happiness on the basis of settlement quality.

## Conclusions and future prospects

### Conclusions

In this study, the link between settlement quality and residents’ happiness is modeled. An IGSA-MLPNN framework, interpreted with the GARSON algorithm, predicts happiness scores from a broad set of urban indicators with high accuracy. The approach captures complex nonlinear relationships and ranks each factor by its influence. The main findings are as follows.

(1) The proposed IGSA-MLPNN model demonstrates strong predictive performance and clear practical value. Compared with traditional linear models, it more effectively captures complex and nonlinear relationships between settlement indicators and happiness. In the case study, the model explains most of the variation in happiness satisfaction and proves feasible for real-world evaluation. These findings indicate that machine learning methods can provide reliable tools for urban residents’ happiness assessment.
(2) The importance analysis indicates that not all indicators contribute equally to residents’ happiness. At the macro level, environmental cleanliness, convenience of basic services, and housing conditions are the most influential factors. At the micro level, cultural and recreational facilities, preservation of historical heritage, social welfare, and street sanitation emerge as key drivers. These indicators reflect residents’ core needs and directly shape their daily experiences; therefore, they are crucial for enhancing residents’ happiness.
(3) The findings provide clear policy implications for urban planning. To enhance overall happiness, efforts should first focus on improving cleanliness, expanding access to daily services, increasing housing affordability, and strengthening social support systems. At the same time, investment in cultural development, historical heritage preservation, and maintenance of public spaces can yield added value. Prioritizing these high-impact areas can help maximize the effectiveness of urban improvement strategies.

### Limitations and future work

Our findings offer the following key insights for urban planning. First, the key quality-of-life indicators we identified provide a basis for governments to formulate urban planning policies. We found that the cleanliness of the urban environment and the accessibility of basic services are the primary drivers of residents’ happiness. Therefore, improving sanitation and daily amenities is essential. Second, housing affordability and the social welfare system are equally important for residents’ happiness, and we therefore urge the government to improve these areas. Finally, although the contribution of cultural and community factors is relatively low, they should be incorporated into long-term planning to enhance residents’ quality of life. However, this study has several limitations:

(1) This study is based on cross-sectional data from a single point in time. However, urban conditions and people’s perceptions are constantly evolving. Future research should consider employing dynamic analytical methods such as time-series analysis, system dynamics, or agent-based modeling. These methods can better capture how policy changes and social developments influence residents’ happiness over time, thereby enhancing the model’s practical value for long-term urban planning.
(2) The results are drawn from one specific city. While some key factors, such as cleanliness and basic services may apply widely, their relative importance can vary across regions. Future studies should test the model in multiple cities or countries. This would help verify which factors are universal and which are context specific. Cross-regional comparisons will also enhance the adaptability and accuracy of policy recommendations.
(3) The model primarily uses urban-level indicators and does not incorporate personal factors such as health, personality, or social connections. Future research could combine individual-level data with city-level indicators to further understand the drivers of residents’ happiness. Furthermore, improving the model’s prediction accuracy is crucial. The use of tools such as SHAP or LIME [83,84] can help clarify the role of individual variables. Policy simulations, such as testing the effect of increasing green space or improving housing conditions, could support more practical and targeted urban policies.

## Supporting information

S1 Table Evaluation index system of human settlement environment perception. (DOCX)
