# Supplementary material for: Modeling the relationship between residents’ happiness and human settlement quality: An IGSA-MLPNN-GARSON approach
Source: PLoS One. 2026 Apr 30;21(4):e0347769. doi: 10.1371/journal.pone.0347769 (PMC13132247; doi:10.1371/journal.pone.0347769)
Supplement: S1 Table — (DOCX) [file pone.0347769.s001.docx]

**S1 Table.** Evaluation index system of human settlement environment perception

| **Target layer** | **Criterion layer** | **Indicator layer** | **Index code** |
| --- | --- | --- | --- |
| Human settlement environment perception | Ecological habitability | Open space | X₁₁ |
|  |  | Noise pollution | X₁₂ |
|  |  | Park convenience | X₁₃ |
|  |  | Hydrophilic space | X₁₄ |
|  |  | Population density | X₁₅ |
|  |  | Water pollution | X₁₆ |
|  |  | Air pollution such as PM2.5 | X₁₇ |
|  | Diversity and inclusiveness | Blind occupation | X₂₁ |
|  |  | House price acceptability | X₂₂ |
|  |  | Rent acceptability | X₂₃ |
|  |  | The standard degree of housing rental market | X₂₄ |
|  |  | Construction of affordable housing | X₂₅ |
|  |  | Urban village reconstruction level | X₂₆ |
|  |  | Friendliness to the migrant population | X₂₇ |
|  | Safety toughness | Disaster warning | X₃₁ |
|  |  | Safety promotion | X₃₂ |
|  |  | Social security | X₃₃ |
|  |  | Traffic order | X₃₄ |
|  |  | Fire safety hazard | X₃₅ |
|  |  | Emergency shelter | X₃₆ |
|  |  | Waterlogging | X₃₇ |
|  |  | Response to disasters | X₃₈ |
|  |  | Access to emergency care | X₃₉ |
|  | Convenient transportation | Road potency | X₄₁ |
|  |  | Riding environment | X₄₂ |
|  |  | Bus waiting time | X₄₃ |
|  |  | Commuting time | X₄₄ |
|  |  | Public transport transfer | X₄₅ |
|  |  | Walking environment | X₄₆ |
|  |  | Car parking convenience | X₄₇ |
|  | Cityscape Features | Urban historic district protection | X₅₁ |
|  |  | Cultural facilities | X₅₂ |
|  |  | The restoration and utilization of historical buildings and traditional residences | X₅₃ |
|  | Healthy and comfortable | Nearby shopping | X₆₁ |
|  |  | Infrastructure | X₆₂ |
|  |  | Shopping mall | X₆₃ |
|  |  | Community elderly care facilities | X₆₄ |
|  |  | Community sports venue | X₆₅ |
|  |  | Infant care service facilities | X₆₆ |
|  |  | Outdoor children's activity facilities | X₆₇ |
|  |  | Nearby primary school enrolment | X₆₈ |
|  |  | Renovation of old residential areas | X₆₉ |
|  |  | Community health service center | X₆₁₀ |
|  |  | Community elderly canteen | X₆₁₁ |
|  |  | Community neighborhood relationship | X₆₁₂ |
|  |  | Electric bicycle charging station | X₆₁₃ |
|  | Neat and orderly | Street hygiene | X₇₁ |
|  |  | Residential property management | X₇₂ |
|  |  | Parking management | X₇₃ |
|  |  | Residential garbage classification | X₇₄ |
|  | Innovation vitality | Youth appeal | X₈₁ |
|  |  | Job opportunity | X₈₂ |
|  |  | Talent introduction policy | X₈₃ |
|  |  | Market environment | X₈₄ |
